# Mechanisms of phosphenes in irradiated patients

**DOI:** 10.18632/oncotarget.18719

**Published:** 2017-06-28

**Authors:** Thibaud Mathis, Stephane Vignot, Cecila Leal, Jean-Pierre Caujolle, Celia Maschi, Martine Mauget-Faÿsse, Laurent Kodjikian, Stéphanie Baillif, Joel Herault, Juliette Thariat

**Affiliations:** ^1^ Department of Ophthalmology, Croix-Rousse University Hospital, 69004 Lyon, France; ^2^ Department of Medical Oncology, Jean Godinot Institute, 51100 Reims, France; ^3^ Department of Ophthalmology, Pasteur II Hospital, 06000 Nice, France; ^4^ Rothschild Ophthalmologic Foundation, 75019 Paris, France; ^5^ Proton Therapy Center, Université Nice Sophia Antipolis, 06200 Nice, France; ^6^ Department of Radiation Therapy, Centre Francois Baclesse, ARCHADE, 14000 Caen, France

**Keywords:** phosphenes, radiation therapy, eye tumors, choroidal melanoma, proton beam therapy

## Abstract

Anomalous visual perceptions have been reported in various diseases of the retina and visual pathways or can be experienced under specific conditions in healthy individuals. Phosphenes are perceptions of light in the absence of ambient light, occurring independently of the physiological and classical photonic stimulation of the retina. They are a frequent symptom in patients irradiated in the region of the central nervous system (CNS), head and neck and the eyes. Phosphenes have historically been attributed to complex physical phenomena such as Cherenkov radiation. While phosphenes are related to Cherenkov radiation under high energy photon/electron irradiation conditions, physical phenomena are unlikely to be responsible for light flashes at energies used for ocular proton therapy. Phosphenes may involve a direct role for ocular photoreceptors and possible interactions between cones and rods. Other mechanisms involving the retinal ganglion cells or ultraweak biophoton emission and rhodopsin bleaching after exposure to free radicals are also likely to be involved. Despite their frequency as shown in our preliminary observations, phosphenes have been underreported probably because their mechanism and impact are poorly understood. Recently, phosphenes have been used to restore the vision and whether they might predict vision loss after therapeutic irradiation is a current field of investigation. We have reviewed and also investigated here the mechanisms related to the occurrence of phosphenes in irradiated patients and especially in patients irradiated by proton therapy for ocular tumors.

## INTRODUCTION

Anomalous visual perceptions such as phosphenes, named from the ancient Greek words *phos* (light) and *phaìnomai* (to show), are visual perceptions of light that occur independently of the physiological and classical photonic stimulation of the retina. Phosphenes can be an early symptom in various diseases of the retina, visual pathways or central nervous system (CNS) although they may also be experienced by healthy individuals in association with emotional factors, drugs, alcohol, stress, fever or psychic conditions [1, 2]. They can be induced by electric or transcranial magnetic stimulation of the visual cortex in sighted and blind subjects [3–5]. Phosphenes have been intensively investigated as a means to restore some vision in the blind [6–8].

They have also been reported by astronauts in space, where high energy particles can interact with the human body [9, 10]. Their true nature in such conditions is still not completely understood. The so-called Cherenkov effect, which shows as a characteristic blue glow resulting from electromagnetic radiations emitted when charged particles pass through the vitreous medium at a speed greater than that of light, is a repeatedly reported explanation for phosphenes in astronauts [10, 11]. Alternate explanations include physiological mechanisms involving retinal stimulation and/or damage (including mitochondrial oxidative processes [12] and bioluminescent ultraweak photons), microgravity [10], or pressure phosphenes [13]. Similarly, irradiated patients commonly report anomalous visual perceptions after therapeutic irradiation of the CNS, head and neck and the eye [14–18]. Phosphenes have been underreported probably because their mechanism and impact are poorly understood. Given that phosphenes have been identified as a means to restore vision, their relevance in irradiated patients is probably underestimated and so are their underlying mechanisms [19–22]. The possibility that phosphenes might predict vision loss after therapeutic irradiation deserves investigation.

We have assessed here the likelihood of radiation interactions with the eye media and alternate mechanisms involved in the occurrence of phosphenes in irradiated patients and also investigated physiological mechanisms based on current knowledge.

### Therapeutic irradiation involving the CNS, head and neck, and the eye

Radiation therapy is frequently used to treat tumors of the CNS, head and neck, and the eye. Concerning ocular tumors, choroidal metastases occur in up to 11% of patients in screening programs for breast or lung cancer patients and are usually treated with conventional radiation therapy [23]. Uveal melanomas are the most common primary ocular tumors with 6 cases per 100.000 inhabitants per year in Western countries and are mostly treated with brachytherapy or proton therapy. Conjunctival tumors and eye lid carcinomas sometimes require radiation therapy as part of their treatment regime. Intra-ocular lymphomas, conjunctival lymphomas and retinoblastomas are very rare malignant tumors of the eye and may require irradiation [24].

Radiation therapy of ocular tumors is challenging because of adjacent sensitive normal tissues and the necessity to destroy the tumor while minimizing the risk of visual loss, dry eye syndrome and glaucoma. Uveal melanomas have five-year local control rates of about 95% and eyeball preservation of about 93% [25–32]. Visual outcomes depend on the initial size and location of the tumor [28–30, 33] but have not yet been correlated with phosphenes. In many other cancers, such as nasopharyngeal and sinonasal tumors or brain metastases and primary CNS tumors, the normal neuro-optic structures receive some dose contribution. The dose contribution varies with the location of the tumor and the eye itself may receive doses of a few Gray (Gy) while optical pathways may receive a dose contribution of up to tens of Gy [23]. Also, with non coplanar beam irradiation such as those in some stereotactic treatments, the radiation beams may cross neuro-optic structures.

## OCCURRENCE OF PHOSPHENES IN IRRADIATED PATIENTS

Phosphenes are frequently reported in patients irradiated in the region of the CNS, head and neck or the eyes regardless of the used irradiation technique. Systematic reporting is rare and thus leads to an underestimation of these symptoms.

### Phosphenes in patients irradiated with electron/photon

Brandes and Dorn in 1896 first reported the production of an X-ray phosphene. When the dark-adapted eye is irradiated with X rays, a homogenous, luminous, blue glow is seen across the visual field. Experiments were conducted in 10 selected cancer patients treated with 6-18 MeV (an electronvolt, or eV, being a measurement unit defined by the kinetic energy acquired by an electron fastened from rest state by a potential difference of one Volt) electrons [18] to an eye in order to assess the occurrence of phosphenes in patients irradiated with electrons. Ten control patients underwent photon/electron radiotherapy to extracranial areas. Both groups had undergone dark-adaptation for 10 minutes before irradiation. All patients who underwent ocular irradiation with electrons experienced blue phosphenes while none of the extracranial cancer patients experienced phosphenes [18].

### Phosphenes in patients irradiated with hadron therapy

Phosphenes can be induced by heavy ion interactions in the eye. Astronauts reported light flashes during the space trip that was part of the moon landing mission. Further space missions confirmed these reports and aimed to detect and correlate cosmic rays with visual sensations [17, 34–36]. The light flash rate was higher going toward the moon than back to earth and also in low earth orbit at high latitudes where particle flux, including protons [37], was higher due to the lower geomagnetic shielding which allows lower energy particles to reach the low earth orbit [36, 38, 39]. The heavy ions and protons used in irradiation therapy are also described as having the ability to produce phosphenes.

#### Phosphenes in patients irradiated with carbon therapy

Phosphenes were investigated, in patients undergoing ocular heavy ion therapy on the eye [17, 40], as a collaborative effort between the ALTEA (Anomalous Long Term Effects on Astronauts) program of the European Space Agency and two medical/physics teams. In clinical practice, patients undergoing ^12^C-ions irradiation for brain tumors, with an energy range between 80-400 MeV/n [41], reported white (90% of cases) or yellow (10% of cases) streak phosphenes quickly moving in vertical or horizontal directions.

#### Phosphenes in patients irradiated with proton therapy

**Figure 1:**
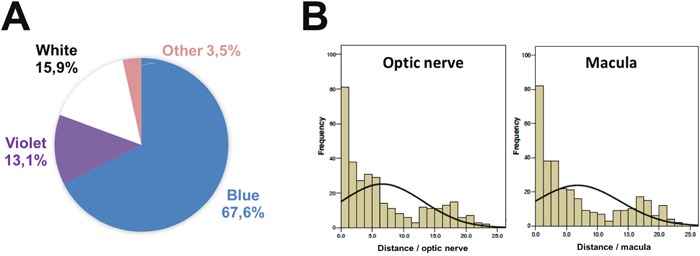
Characterization of phosphenes in patient irradiated by proton beam for eye tumor **(A)** Color of phosphenes. **(B)** Occurrence of phosphenes depending on distance to optic nerve (left) and macula (right).

Proton therapy is used in ocular oncology to treat conjunctival or uveal tumors such as hemangioma, metastases and melanomas [25, 42–44]. Two-thirds of patients undergoing ocular proton therapy for choroidal melanoma reported phosphenes when over 20% of their retinal surface received a high dose [16, 45]. On average, patients who perceived phosphenes reported about three light flashes per session [45]. A large majority (73.8%) of the flashes were described as a blue light. In this study, the more the optic disc was irradiated, the more the light flashes were observed [45]. In the latter condition, these light flashes were less frequently blue. In our experience of 229 patients questioned immediately after ocular proton therapy, 70% of these dark-adapted patients treated for choroidal melanomas reported phosphenes versus 66% of those treated for hemangiomas, 28% for conjunctival tumors and none for iris melanomas (Table 1). The median total dose was 52 Gy with 1.3%, 8.3% and 90.4% of patients respectively receiving low (17 Gy), intermediate (45 Gy) and high doses (52 Gy) corresponding to a hemangioma, conjunctival tumor or uveal melanoma. Patients had blue, violet, white or yellow/orange/green phosphenes respectively in 67.6%, 13.1%, 15.9% and 3.5% of cases. Blue/violet phosphenes (of similar wavelength characteristics) represented 80.7% of cases (Figure 1A). The shapes of the phosphenes were variable. There was no significant impact of the dose level on the perception of phosphenes. In choroidal melanoma, 61% of the patients over 65 years of age had phosphenes of which blue was 70% compared with 76% of 40-65 years old patients and 93% of patients younger than 40 years (p=0.002). The occurrence of phosphenes also correlated with the distance from the tumor to the fovea and the optic disk (p<0.05). The perceived color of the phosphenes correlated with the distance from the tumor to the fovea; this correlation was stronger for a spectrum of colors involving the cones, white excluded (p<0.05). The white phosphenes correlated with the distance from the tumor to the optic disk (p<0.05) (Figure 1B). Phosphene color was unrelated to age, percent of retinal surface irradiated, tumor diameter, thickness or margins and the presence of natural lens/pseudophakia. Unlike proton therapy, no study reported phosphene in irradiated patient by eye plaque brachytherapy.

**Table 1:** Occurrence of phosphene depending on tumor localization

Tumor irradiated	Frequency
Choroidal melanomas	70%
Hemangiomas	66%
Conjonctival tumors	28%
Iris melanomas	0%

## MECHANISMS OF PHOSPHENES

### Mechanisms of phosphenes in non-irradiated patients

Phosphenes were first described in ophthalmology by Moore in 1935 when investigating rupture of the peripheral retinal cysts [46]. Phosphenes have since been associated with various eye-related conditions such as posterior vitreous detachment, retinal detachment, retinal break or tear, focal infections, and choroidal tumors [2]. In these cases, phosphenes have been shown to be the result of a mechanical stimulation of some cells of the retina secondary to tension or traction applied to the retina.

Extra-retinal stimulation can also lead to phosphenes, with the transduction message starting directly from the optic tract, such as the optic chiasm and optic nerve depending on the stimulation area. As shown by Whilhelm-Buchstab et al., a depolarization process in neural tissues or free radicals in the glia, both induced by irradiation, can directly or indirectly induce message transduction within the optic tract, from the irradiated area to the occipital brain [47]. This may explain phosphenes in degenerative lesions of the nerves and brain (retro-bulbar optic neuropathy, trauma, sclerosis…) where the nerve fibers can more easily be depolarized or affected by calcium currents.

Phosphenes can also occur in up to 90% of healthy patients having migraines with aura [48]. Associated visual phenomena include negative (blind spots or scotomas) or positive symptoms (scintillating scotoma and photopsia) [49]. Using transcranial magnetic stimulation (TMS), so-called “magnetophosphenes” are more frequently generated in patients with migraines and aura rather than controls [49], and at lower energy thresholds than in patients without aura [50]. These studies show hyperexcitability of the visual tracks in patients suffering from migraine with aura [49, 51]. Systemic diseases such as thyroid eye disease can also result in phosphenes [52] through mitochondrial respiration chain and lipid peroxidations resulting in light emitting molecules in the retina [53, 54].

### Mechanisms of phosphenes in irradiated patients

#### Cherenkov effect

The first historically considered mechanism responsible for phosphenes is Cherenkov radiation. In a material different than vaccum such as the vitreous when considering the eye, it becomes possible for an accelerated particle to acquire a velocity higher than the phase velocity of light in that medium. The super-fast particle creates a “shockwave” when becoming faster than the phase velocity of light (an analogy can be made with a plane going faster than the speed of sound).

**Figure 2:**
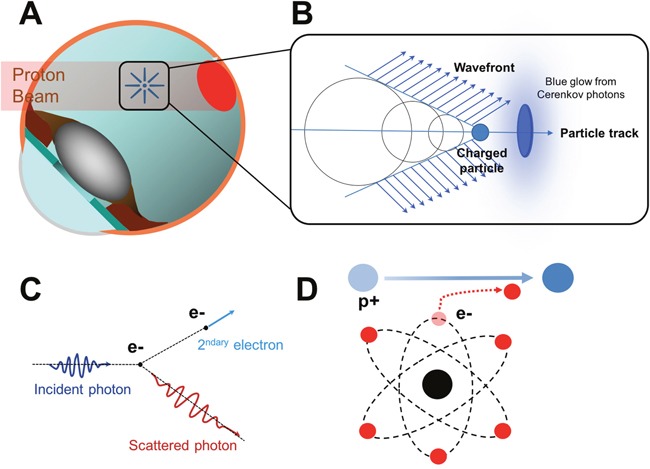
Role of the Cherenkov radiation in the occurrence of phosphenes in patients irradiated by proton therapy **(A)** Schematic diagram showing the irradiation of a choroidal tumor (in red) by the proton beam. **(B)** Cherenkov effect. The charged particle is moving faster than the waves it emits. **(C)** Representation of a Compton scattering. Inelastic scattering of a photon by an electron results in decreasing energy of the photon; A part of the energy is transferred to the electron. **(D)** Representation of a Coulomb interaction of a proton with an atomic electron. The opposite charges of protons and electrons cause an attraction of the atomic electron (in red) by the proton (in blue).

When a charged particle moves through a medium at a speed higher than the speed of light in that medium, a faint radiation is produced by the medium. In water, for example, the charged particle excites the water molecules which then return to their normal state by emitting photons of blue light. It can trigger a cascade of photons that are in phase with each other and can interfere constructively to form a visible blue glow. When the particle goes faster than the waves it emits, the waves are found behind the incident particle and are included in a cone whose top is the moving particle (Figure 2A, 2B). Cherenkov radiation produces a broad continuous spectrum of light emission from UV down to near-infrared, with a spectrum described mathematically by the Frank-Tamm formula [55] which predicts the number of photons emitted within a wavelength interval. The higher the particle energy, the stronger the photon emission with optical emission predominating in the UV blue range [15]. Typical Cherenkov radiation is emitted as a conical surface, perceived as a cone of blue-violet light [56, 57].

#### In conventional electron/photon radiation therapy

In patients irradiated with high-energy electrons, Cherenkov radiation is assumed to be the dominant mechanism for phosphenes due to the consistently reported cone/ring shaped blue light in these patients. The calculated threshold energy of electrons to reach a sufficient velocity to produce a Cherenkov light is 0.264 MeV in water and 0.219 MeV in tissue (using a refractive index of 1.4) [55]. Further experiments, using water phantoms and a camera coupled to an optical fiber, investigated whether Cherenkov emissions occurred during photon (6 and 18 MV) and electron (6, 9, 12, 15, 18 MeV) irradiations [15].

Photons produced phosphenes owing to a Cherenkov emission occurring through secondary electrons produced by photoelectric or Compton effects [14] (Figure 2C). The threshold photon energy required to produce Compton electrons which exceed the Cherenkov threshold energy (0.264 MeV) is 0.422 MeV [55]. The published literature concerning high energy photon and electron beam irradiation consistently identifies Cherenkov radiation as the most likely cause of phosphenes in patients undergoing intracranial/eye irradiation [18, 56, 58].

#### In carbon therapy

The threshold energy for heavy ions to directly produce Cherenkov light is 482 MeV in water [55]. The therapeutic ion beam energy is below the Cherenkov threshold and so phosphenes could not be attributed to Cherenkov radiation but to beam-spot impact near the rear part of the eyes [41]. As phosphenes were observed by all patients who were treated with an eye-directed field and with a lower frequency by those treated with a field very close to the eye where some scattered particles may have been present, phosphenes were attributed in these patients to direct energy deposition by charged particles onto the retina [40, 41]. The perception of predominantly white phosphenes was not further explained.

#### In proton therapy

Calculations showed that in ocular proton therapy the treatment energy is far below the threshold of 482 MeV/n with n as refractive index (the maximum energy used to treat ocular tumors is about 65 MeV). This rules out the possibility for protons and heavy ions to directly induce phosphenes by a Cherenkov effect. A proton beam loses its energy mainly by coulomb interactions with electrons [55] which lead to ionization and the liberation of secondary electrons (Figure 2D). If these secondary electrons have energy exceeding 0.262 MeV, they will emit Cherenkov radiation. A proton beam of 120 MeV or higher energy is needed in order to produce secondary Coulomb electrons which exceed this threshold [59]. In such a case, the Cherenkov effect might explain phosphenes. However, phosphenes are also described by patients undergoing ocular proton therapy at effective energies below 65 MeV [16]. The proton energy after the range shifter and modulator wheel (two accessories placed along the proton beam to degrade proton energy in order to produce a spreadout bragg peak at the tumor depth) is typically a few tens of MeV [16] and secondary electrons have energies below the threshold for production of the Cherenkov effect. However, Helo et al. recently suggested that Cherenkov emission might be due to indirect emissions of fast electrons liberated by prompt gamma and neutron emission, or a slow component from positrons and electrons emitted by radioactive decay [59].

#### In space

The phosphenes reported by astronauts were investigated for a Cherenkov effect [9]. Studies were performed during the Apollo missions to detect and correlate cosmic rays with visual sensations [36]. The light flash rate was higher going towards the moon than back to earth and also in low earth orbit at high latitudes where particle flux, including protons [37], was higher due to the lower geomagnetic shielding which allows lower energy particles to reach the low earth orbit [36, 38, 39]. It was later demonstrated that the probability of phosphenes increased with Linear Energy Transfer and that nuclei and largely ionizing particles were the dominant sources of phosphenes in space [17, 39, 60]. However, it remains unclear how energy deposition by ionizations gets transformed into a light signal to the brain [17]. The ALTEA program [61] investigated whether ions entering the eye can couple directly with the sensorial pathway, and/or produce photons, which act as transducers to turn ion energy into a signal for the visual sensors in the retina [61, 62]. Using electroencephalographs and particle tracking in space and on the ground [62], phosphenes could not be fully explained by differences in radiation type and fluence suggesting a role for physiological parameters [15–18].

Overall, the published literature concerning irradiation with heavy ions and protons appeared to indicate that phenomena other than Cherenkov radiation are responsible for phosphenes. As phosphenes are very similar during heavy ions irradiation and in space, conclusions from a clinical series of irradiated patients and data from space programs are important to unravel the underlying phenomena for phosphenes and its visual consequences.

#### Direct stimulation of the retina

**Figure 3:**
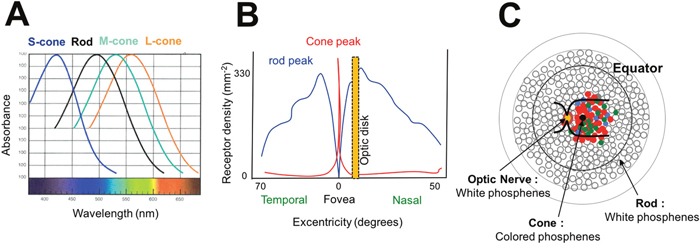
Hypothesis of direct stimulation of the retina **(A)** Wavelengths absorbed by rod and cone chromophores. Short, Medium and Long wavelengths are respectively absorbed by blue (S-cone), green (M-cone) and red (L-cone) cones. **(B)** Distribution of cones and rods in the retina. Cones are mainly located in the foveal area while rods are predominantly located in the optic nerve area and the retinal periphery. **(C)** Mechanisms of phosphenes according to the irradiated area. If cones are stimulated (macular area), colored phosphenes are predominant. If rods are stimulated (optic nerve and retinal periphery), white phosphenes are predominant.

Phosphenes can result from a photochemical breakdown in the rods and cones by Photons X and Gamma [63, 64] and there is a relation between amplitude on the electroretinogram and surface of exposed retina [65, 66]. The importance of photopigment bleaching has also been linked to energy transfer from the opsin to the attachment site of the chromophoric group [65, 66]. Patients undergoing ocular proton therapy experience colored phosphenes that correlate with proximity to the macula and exhibit strikingly blue predominance in a mesopic ambience. The spectrum of visible light for the human eye is composed of a multitude of wavelengths between 400 and 700 nm depending on the opsin forming the cone's chromophore. It includes short wavelengths perceived as violet to blue (400-500 nm) mediated by S-cones, intermediate wavelengths perceived as green to orange yellow (500-600 nm) mediated by M-cones and long wavelengths perceived as yellow to red (550-700 nm) mediated by L-cones [67] (Figure 3A). Cones are mainly located in the macular area and in the fovea [68, 69] and may be associated with production of colored phosphenes (Figure 3B). On the other hand, rods’ chromophores can absorb photons, induce changes in rhodopsin and end-up activating a non-colored transduction pathway (black and white). They are located in the peripheral retina and are involved in scotopic and mesopic vision [68, 69]. White phosphenes, second in frequency, could result from the activation of cones (from maximal desaturation of elementary colors) but also rods, particularly as a correlation was noted with the distance to the optic nerve around which the rods’ density is high [45] (Figure 3C).

#### Ultraweak bioluminescent photons

Spontaneous and induced ultraweak bioluminescent photons may also explain the occurrence of phosphenes independent of the Cherenkov radiation [54, 70]. Ultraweak bioluminescent photons are particles permanently present at a low concentration within the eye and brain [70, 71]. They are generated by cells in physiological conditions when these emit free radicals essentially derived from the mitochondrial respiration chain and lipid peroxidation metabolism [72, 73]. The oxidation of biomolecules initiates a cascade of oxidative reactions that lead to the formation of electronically excited species which, in turn, can emit a photon [74].

The above two physiologic mechanisms can be altered or enhanced in various situations resulting in a higher local concentration of free radicals and ultraweak bioluminescent photons. Phosphenes may also be caused by the production of ultraweak biophotons by the CNS cells in the visual system secondary to local overproduction of free radicals from ionizing radiation [54], as in space [75, 76]. *In vitro* water radiolysis (water being a major component of the human body) produces radicals near retinal photoreceptors (rods) and further chemically interacts with lipids (peroxidation) to generate chemiluminescence emitting photons in the visual spectrum [76].

**Figure 4:**
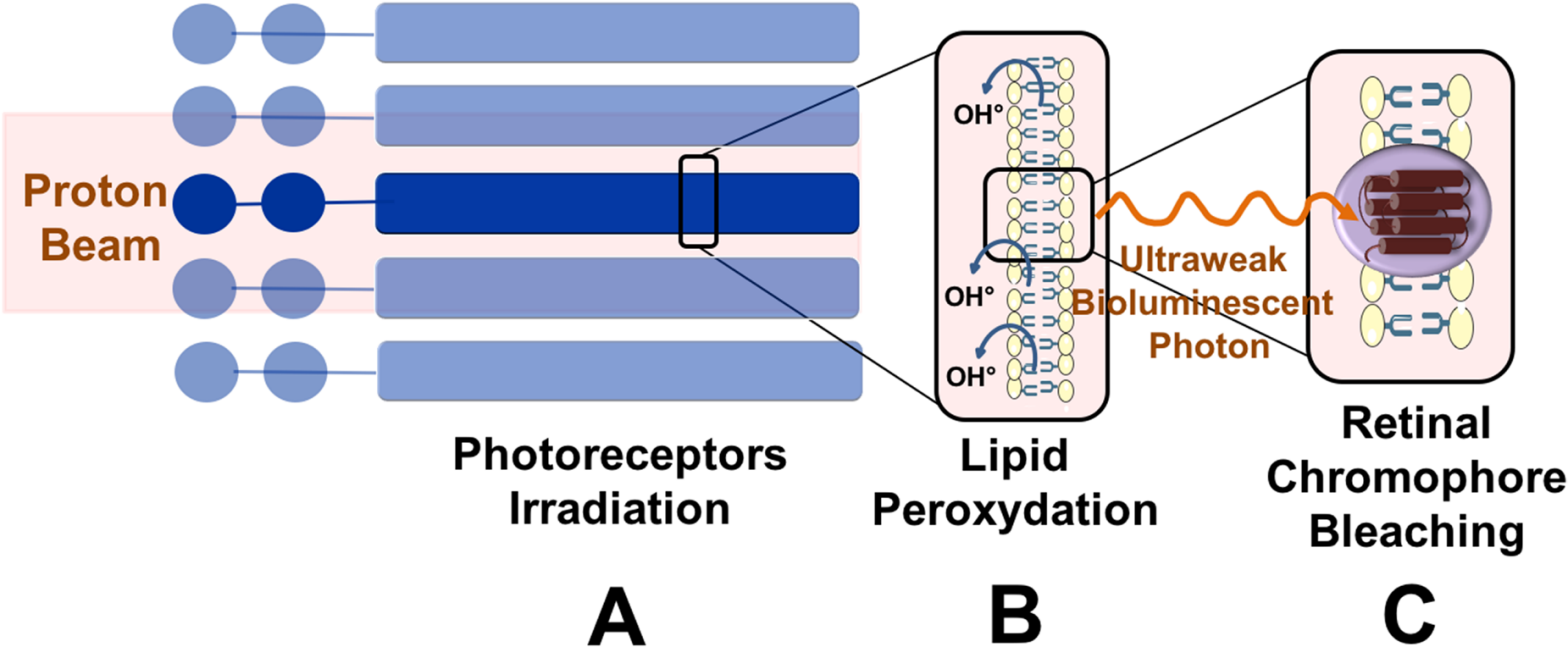
Role of ultraweak bioluminescent photon emissions **(A)** Direct irradiation of photoreceptors by the proton beam. Photoreceptor outer segments are directly irradiated by the proton beam. **(B)** Lipid peroxidation of the photoreceptor outer segments and production of free radicals. Free radicals (OH°) are produced secondary to ionizing irradiation near or within the retina. Free radicals react with lipids of the phospholipidic membrane and result in lipid peroxidation. Chemical interactions result in ultraweak bioluminescent photons by free radicals. **(C)** Absorption of ultraweak bioluminescent photons by retinal chromophores leading to the perception of phosphenes. The ultraweak bioluminescent photons bleach the retinal chromophores activating rods and cones. The subsequent phototransduction cascade results in the perception of phosphenes.

Therapeutic irradiation can create specific conditions which enhancing the number or perception of ultraweak bioluminescent photons. Indeed, nuclear interactions secondary to protons on the retina could also result in the production of an excess of free radicals in the eye [76–78], similar to that with heavy ions (200 MeV/n ^12^C to doses of 10 to 100 mGy) [79]. This process is dependent on the local concentration of anti-oxidants which is subject to inter-individual variations [9]. Over a threshold concentration, ultraweak bioluminescent photons can be absorbed by photoreceptors and bleach the rhodopsin molecules inducing a photo-transduction cascade that finally produces photopic signals interpreted by the brain as phosphenes [54, 71] (Figure 4). Intact bovine rod outer segments in suspension were irradiated with ^12^C and rhodopsin was bleached with a linear dose/effect relationship although without apparent functional damage [17, 40, 76, 80]. Rhodopsin bleaching was proportional to the concentration of the radical concentration and full regeneration was possible. Polyunsaturated fatty acids surrounding the rods can propagate free radicals initially generated by peroxidation [40]. Inter-individual variations in the perception of phosphenes may further depend on individual conditions (stress, diet, free radicals concentration, detoxifying enzymes …) [76]. The wavelength of the ultraweak photon emission (UPE) is measured in nanometers. Some of the research on ultraweak photon emissions reported UPE from 420 to 570 nm [73]. This range corresponds to the visible light color ranges from mainly violet or blue to green. Then, the biophotons may be absorbed by the photopigment of the photoreceptor to stimulate the visual pathway resulting in phosphenes. If the wavelength of the biophoton is near the blue spectrum, a blue flash may occur after absorption by the blue opsin (S-cone).

#### Intrinsically photosensitive retinal ganglion cells (ipRGC) stimulation

Another explanation for phosphenes in irradiated patients could involve melanopsin cells. Melanopsin, a light sensitive pigment [81], is present in retinal ganglion cells (0.3% of all ganglion cells) which regulate non-visual functions such as the circadian cycle and pupillary constriction [82]. These specific ganglion cells, called “intrinsically photosensitive Retinal Ganglion Cells” (ipRGC), may also behave as independent photoreceptors [83]. The ganglion cell layer consists of large 10-20 μm thick neural cells. It is almost exclusively a monolayer, except around the fovea where 7-8 ranks of nuclei are superimposed.

**Figure 5:**
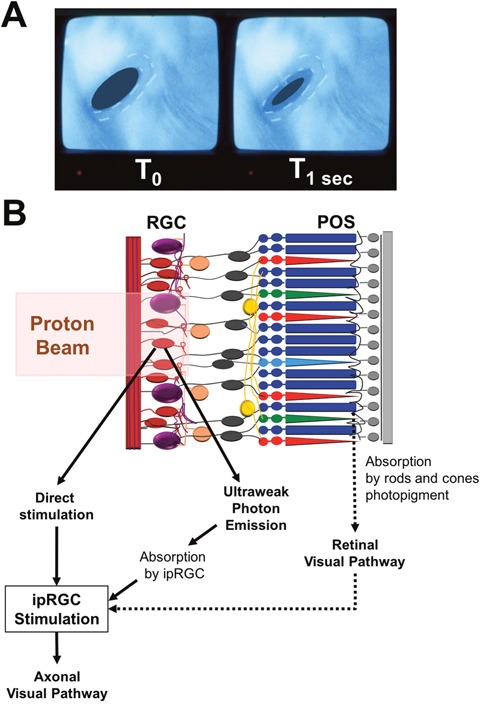
Role of intrinsically photosensitive retinal ganglion cells in radiotherapy-induced phosphenes **(A)** In-room camera view shows pupillary constriction between left (T_0_) and right (T_1 sec_ after initiation of the proton beam) inserts. **(B)** Schematic diagram showing the interactions between retinal ganglion cells, intrinsically photosensitive Retinal Ganglion Cell (ipRGC) and the proton beam. There are 3 hypotheses concerning the activation of ipRGC: (1) Direct stimulation by the proton beam. (2) Production of ultraweak bioluminescent photons which are then absorbed by the ipRGC. (3) Stimulation of rods and cones photopigments in the photoreceptor outer segments (POS) and activation of ipRGC by the normal retinal visual pathway. When excited, ipRGC discharges nerve impulses which travel through neuronal axons to specific brain targets such as the center for pupillary control.

Pupillary constriction at the initiation of proton beam therapy occurs in half the patients experiencing phosphenes but seldom in the patients without phosphenes [84]. This observation suggests the involvement of ipRGC in the occurrence of phosphenes. Such interactions between different retinal cells illustrate the complexity of the retinal physiology in response to radiation therapy (Figure 5A).

The closer the irradiated area is to the optic disk and fovea (most ganglion cells are next to the fovea and predominate in the nasal and peripheral areas), the more relevant this hypothesis concerning the involvement of ipRGC in the causation of phosphenes. Their intrinsic photoactivation threshold is high and is further increased upon stimulation by short wavelengths; this intensity is higher than with S-cones [85]. Ganglion cell involvement in the origin of blue phosphenes cannot be confirmed because cones, rods and ipRGC all participate in the pupillary reflex and the involvement of individual cells cannot be evaluated [86]. Recent studies suggest that ipRGC could also be involved in the production of phosphenes [87, 88]. Retinal ganglion cells can convey spatial information in advanced retinal degeneration [89]. Further, similar to living cells [72, 90], retinal ganglion cells continuously emit ultraweak bioluminescent photons without external excitation. These biophotons can then be absorbed by the photopigments of retinal ganglion cells, triggering retinal waves or sending signals to the retinotopic superior colliculus [91] (Figure 5B).

## CONCLUSIONS AND FUTURE DIRECTIONS

Phosphenes in irradiated patients are probably underestimated due to lack of understanding of their mechanisms and relevance to vision. They are due to specific mechanisms depending on the type of irradiation and structures involved; particle physics interactions, stimulation of photoreceptors, free radicals or ultraweak bioluminescent photons may be involved depending on complex and specific conditions. Most importantly, as suggested by the investigations on phosphenes and retinal prostheses as a means to restore vision, it is likely that not only can phosphenes unravel important physiological mechanisms in irradiated patients but may also help personalize their treatments and better predict visual outcomes.
